# Prognostic significance of compliance with fractional flow reserve guidance on diverse vessel-related clinical outcomes

**DOI:** 10.3389/fcvm.2024.1370345

**Published:** 2024-05-17

**Authors:** Yang Zhang, Bangguo Yang, Yicong Ye, Xiliang Zhao, Yaodong Ding, Yi Ye, Liang Zhang, Dawei Tan, Gong Zhang, Xiaoyu Duan, Quan Li, Yong Zeng

**Affiliations:** ^1^ Center for Coronary Artery Disease, Division of Cardiology, Beijing Anzhen Hospital, Capital Medical University, Beijing, China; ^2^ Department of Cardiology, Fuwai Yunnan Cardiovascular Hospital, Kunming, China; ^3^ Division of Cardiology, Beijing Daxing District People’s Hospital, Beijing, China; ^4^ General Medicine Department, The Central Hospital of Wuhan, Tongji Medical College, Huazhong University of Science and Technology, Wuhan, China

**Keywords:** coronary artery disease, fractional flow reserve, percutaneous coronary intervention, vessel-oriented composite endpoints, target vessel failure

## Abstract

**Background:**

In patients underwent fractional flow reserve (FFR) assessment, a noteworthy proportion of adverse events occur in vessels in which FFR has not been measured. However, the effect of these non-target vessel-related events on the evaluation of FFR-related benefits remains unknown.

**Methods and results:**

In this retrospective study, vessels subjected to FFR measurement were grouped as FFR-based approach and non-compliance with FFR based on whether they received FFR-based treatment. Using inverse probability of treatment weighting (IPTW) to account for potential confounding, we investigated the association between compliance with FFR and 5-year target vessel failure (TVF) non-target vessel failure (NTVF) and vessel-oriented composite endpoints (VOCEs). Of the 1,119 vessels, 201 did not receive FFR-based treatment. After IPTW adjustment, a signiﬁcantly lower hazard of TVF was observed in the FFR-based approach group (HR: 0.56; 95% CI: 0.34–0.92). While, the intergroup difference in hazard of NTVF (HR: 1.02; 95% CI: 0.45–2.31) and VOCEs (HR: 0.69; 95% CI: 0.45–1.05) were nonsignificant.

**Conclusions:**

In patients with CAD subjected to FFR, the FFR-based treatment yields a sustained clinical benefit in terms of the risks of target vessel-related events. The dilution of non-target vessel-related events renders the difference favoring the FFR-based approach nonsignificant.

## Introduction

The potential benefits of revascularization in patients with coronary artery disease (CAD) depends upon the presence of inducible myocardial ischemia. Therefore, it is crucial to accurately identify whether coronary stenoses are associated with impaired coronary flow (1, 2). As the standard method for invasive coronary physiological assessment, fractional flow reserve (FFR) evaluates the impact of stenosis on coronary flow by measuring the pressure gradient across the lesion (3). Incorporating FFR in decision-making for percutaneous coronary intervention (PCI) has demonstrated improves clinical outcomes compared with the angiography-guided strategy (4, 5).

Establishing the myocardial revascularization strategy is a comprehensive decision-making process, with a multitude of factors beyond the FFR index potentially influencing the establishment of the treatment plan. Previous studies have revealed that a subset of cases did not adherent to FFR measurements in their treatment strategies (5, 6). Nevertheless, the generalizability of these findings to an all-comer population in real-world clinical practice remains uncertain. Additionally, given its remarkable spatial resolution for identifying ischemia-related stenoses, the benefits of the FFR-based strategy are primarily apparent at the vessel level. Data from previous registry suggest that 22.3% of adverse cardiovascular events in patients undergoing FFR measurements are, in fact, attributed to lesions that were not subjected to FFR measurements (2). If the attribution of vessel-oriented composite events is not well-defined, the dilution effect of non-target vessel adverse events could potentially preclude the proper assessment of clinical benefits for FFR-measured vessels. This might partly explain the inconsistency in the conclusions concerning the clinical benefit of the FFR-based approach in studies involving patients with multivessel coronary disease (7–10). Nevertheless, there exists a scarcity of evidence concerning the impact of non-target vessel-oriented events on the appraisal of FFR-related benefits. In this regard, the present study aimed to evaluate the correlations between FFR-based treatment and diverse vessel-oriented adverse events.

## Methods

### Study population

Adult patients with at least one angiographically identified lesion with 50%–90% stenosis in a major epicardial coronary artery or major side branch with a vessel diameter of ≥2.25 mm, followed by successful FFR measurement of the target vessel, were consecutively screened for enrollment. The major criteria for study exclusion were as follows: left main CAD, chronic total occlusion, graft lesions, cardiogenic shock, Thrombolysis in Myocardial Infarction (TIMI) flow of <2, planned coronary artery bypass grafting after diagnostic angiography, and limited life expectancy due to comorbidity. Recent acute myocardial infarction (MI) occurring more than 5 days prior to the index FFR measurement was not an exclusion criterion in this study. The study protocol was in accordance with the Declaration of Helsinki and was approved by Ethics Committee of Beijing Anzhen Hospital, Capital Medical University (No. 2023100X). Written informed consent was obtained from individual or guardian participants.

### Study procedure

The physiological index measurement was performed using a pressure wire (Certus, Abbott Vascular or Prestige, Philips Volcano) placed at the distal end of the target vessel during maximum coronary hyperemia. Hyperemia was achieved by intravenous administration of adenosine (140–180 µg/kg/min). FFR measurement was obtained by calculating the ratio of mean distal coronary pressure to mean aortic pressure. Stenosis with an FFR of 0.8 or less was regarded as positive for ischemia. Further therapy strategy for the patients, according to routine clinical practice, was at the discretion of the treating physician. The FFR-based approach was defined as performing PCI for vessels with ischemic lesion (FFR ≤ 0.8) and deferral of PCI for the vessels with nonischemic lesion (FFR > 0.8), otherwise referred to as non-compliance with FFR.

### Endpoints and definitions

The objective of this study was to elucidate the relationship between FFR compliance and 5-year clinical outcomes on vessel-level. Target vessel failure (TVF) was defined as the combination of cardiac death, target vessel-related myocardial infarction (TVMI), and unplanned target vessel revascularization (TVR), as determined from the date of FFR measurement. All cardiac deaths were considered target vessel-related unless an unequivocal non-target vessel cause could be established. TVMI was defined as MI driven by any lesion located in the same epicardial vessel with an index FFR measurement. The diagnosis of MI was determined according to the third universal definition of MI (11). TVR was defined as a reintervention driven by any lesion in the same epicardial vessel. Any vessel-oriented MI or revascularization occurring in the coronary arteries not assessed by FFR was considered non-target vessel failure (NTVF). The definition of vessel-oriented composite endpoints (VOCEs) was the combination of TVF and NTVF. The vessel-related endpoint without a clearly identifiable culprit vessel was by default attributed to the vessel with the lowest FFR. Event adjudication was conducted by an independent clinical events committee whose members were blinded to both the measured FFR values and treatment.

### Data collection and clinical follow-up

At the time of the initial physiological assessment, clinical baseline data and procedural details were recorded and archived using a dedicated electronic case report system. Clinical follow-up was conducted through outpatient clinic visit or telephone contact with patients or their relatives at 12, 24, 36, 48, and 60 months after FFR measurement.

### Statistical analysis

Baseline characteristics and clinical endpoints were assessed on a per-vessel basis. Discrete variables are presented as counts and percentages, and were compared between groups with the use of chi-square test or Fisher's exact test as appropriate. Continuous variables are presented as means with standard deviations or medians with interquartile range, and were compared using Student's *t*-test or Mann-Whitney *U* test, depending on the data distribution. The distribution normality of continuous variables was carried out using Kolmogorov-Smirnov test and visual inspection of Q-Q plots. Multiple imputation was used to impute missing values for left ventricular ejection fraction in 118 datasets. Outcomes throughout the follow-up were visualized by using Kaplan-Meier time-to-event plots, and the treatment groups were compared using the log-rank test. Patients were censored from the time-to-event analyses when any prescribed endpoint event occurred, or at the time at which they were last known to be alive. Post-hoc landmark analyses were carried out 12 months subsequent to the initial FFR measurement, including only those patients who remained event-free at the 12 months. A hazard ratio (HR) with corresponding 95% confidence intervals (CIs) were derived from marginal Cox proportional hazard regression that to take into account for clustering of multiple vessels within patients.

To consolidate the ﬁndings, the inverse probability of treatment weighting (IPTW) analysis which based on propensity scores was performed. The propensity for being in each group was estimated using a logistic regression model, with covariates including sex, hypertension, peripheral vascular disease, previous cerebrovascular disease, prior PCI, types of acute coronary syndrome, left ventricular ejection fraction, aspirin therapy status, target vessel, multivessel disease, lesion length, pre-procedure FFR value, diameter stenosis, lesion calciﬁcation, bifurcation lesion, pre-procedure TIMI flow grade, lesion with aneurysm, and lesion with myocardial bridge. Differences between groups were measured using standardized differences, where a threshold of 10% indicated a meaningful difference in the covariates. To estimate the between-group differences, three models were fitted: (1) a marginal Cox proportional-hazards regression model; and (2) a multivariate-adjusted model with IPTW. Test for proportional hazards assumptions were based on the scaled Schoenfeld residuals. We conducted subgroup analyses under the same proportional hazards model as in the primary analysis, and the results were presented in a forest plot. Additionally, we performed a sensitivity analysis to test the robustness of the results, excluding vessels with ostial and tortuous lesions, TIMI flow grade of <3, and those from patients with MI, since these factors may affect the FFR measurements accuracy. *P*-values were calculated two-sided with a level of 0.05 indicating statistical significance. Statistical analyses were performed using R 4.2.1 (R Foundation for Statistical Computing).

## Results

### Baseline clinical characteristics

**Figure 1:**
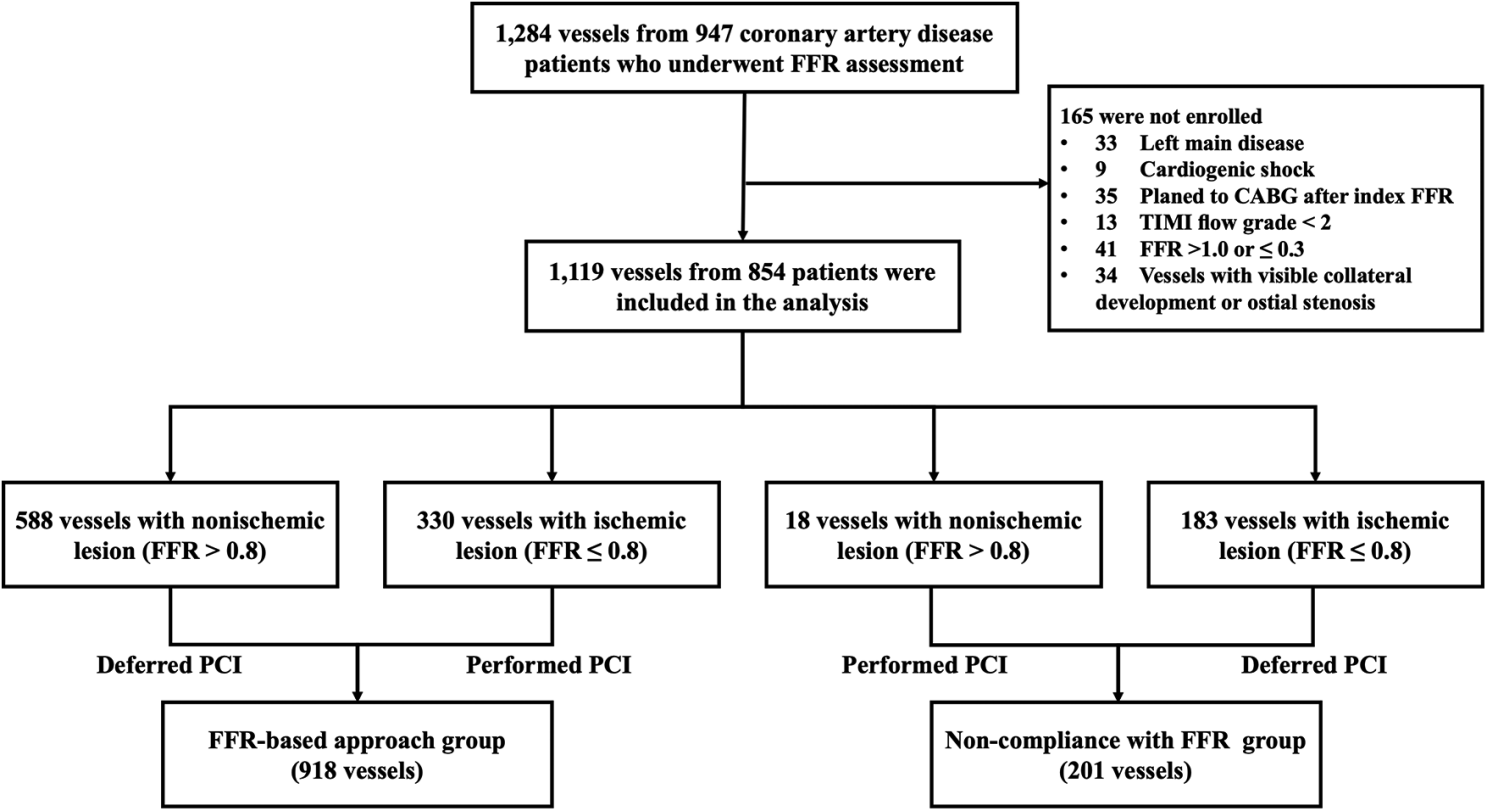
Study flow diagram. CABG, coronary artery bypass grafting; FFR, fractional ﬂow reserve; PCI, percutaneous coronary intervention; TIMI, Thrombolysis In Myocardial Infarction.

A total of 1,119 vessels from 854 patients were analyzed in this study (Figure 1). The mean age of the patients was 58.9 ± 9.0 years, with 73.7% being male. Diabetes mellitus was present in 39.7% of the patients, and 81.0% presented with unstable angina (Table 1). The overall mean FFR value of vessels was 0.80 ± 0.10. Baseline characteristics of vessels among groups are detailed in Table 2. The majority of the investigated vessels were left anterior descending arteries, representing 58.3% of the vessels in the FFR-based group and 72.1% of the vessels in the non-compliance with FFR group, respectively. The differences in baseline variables between the groups were attenuated following IPTW adjustment (Table 2).

**Table 1:** Baseline characteristics of patients.

Patients	*n* = 854
Demographics	
Age, yrs	58.9 ± 9.0
Male	629 (73.7)
BMI, kg/m^2^	26.0 ± 3.0
Medical history and risk factors	
Current smoking	260 (30.4)
Family history of coronary artery disease	104 (12.2)
Left ventricular ejection fraction, %	64.6 ± 6.8
Clinical indication	
Stable angina	116 (13.6)
Unstable angina	692 (81.0)
NSTEMI	26 (3.0)
STEMI	20 (2.3)
Diabetes mellitus	339 (39.7)
Hypertension	580 (67.9)
Dyslipidemia	104 (12.2)
Previous myocardial infarction	70 (8.2)
Previous PCI	95 (11.1)
Previous CABG	4 (0.5)
Previous cerebrovascular disease	49 (5.7)
Peripheral artery disease	35 (4.1)
Chronic kidney disease	7 (0.8)
Medications	
Aspirin	805 (94.3)
Statin	799 (93.6)
ACE inhibitor/ARB	407 (47.7)
Beta-blocker	478 (56.0)
Calcium-channel blocker	212 (24.8)

Values are mean ± SD or *n* (%).

ACE, angiotensin-converting enzyme; ARB, angiotensin receptor blocker; BMI, body mass index; CABG, coronary artery bypass graft surgery; NSTEMI, non-ST-elevation myocardial infarction; PCI, percutaneous coronary intervention; STEMI, ST-elevation myocardial infarction.

**Table 2:** Baseline characteristics of the vessels.

	Unmatched				IPTW			
	Compliant to FFR		*p* value	Standardized difference	Compliant to FFR		*p* value	Standardized difference
	No (*n* = 201)	Yes (*n* = 918)			No (*n* = 188.63)	Yes (*n* = 920.46)		
Lesion location			0.002	0.345			0.141	0.234
Left anterior descending artery	145 (72.1)	535 (58.3)			117.7 (62.4)	556.1 (60.4)		
Left circumﬂex artery	23 (11.4)	129 (14.1)			24.1 (12.8)	125.7 (13.7)		
Right coronary artery	20 (10.0)	190 (20.7)			25.8 (13.7)	180.3 (19.6)		
Diagonal branches	10 (5.0)	41 (4.5)			16.0 (8.5)	37.5 (4.1)		
Obtuse marginal branch	3 (1.5)	23 (2.5)			5.0 (2.7)	20.9 (2.3)		
Diameter stenosis, %	71.69 ± 8.07	70.68 ± 10.18	0.185	0.111	72.03 ± 7.79	70.96 ± 10.21	0.113	0.118
Lesion length			<0.001	0.336			0.636	0.082
<10 mm	22 (10.9)	130 (14.2)			26.3 (14.0)	120.6 (13.1)		
10–20 mm	71 (35.3)	446 (48.6)			81.1 (43.0)	433.1 (47.1)		
>20 mm	108 (53.7)	342 (37.3)			81.2 (43.1)	366.7 (39.8)		
Ostial lesion	23 (11.4)	96 (10.5)	0.776	0.032	22.3 (11.8)	94.9 (10.3)	0.556	0.048
Bifurcation lesion	17 (8.5)	54 (5.9)	0.231	0.100	15.8 (8.4)	59.5 (6.5)	0.397	0.073
Tortuous lesion	4 (2.0)	10 (1.1)	0.490	0.073	2.6 (1.4)	11.5 (1.2)	0.856	0.013
Vessel with myocardial bridge	1 (0.5)	26 (2.8)	0.089	0.183	3.1 (1.6)	22.1 (2.4)	0.701	0.054
Moderate-to-severe calciﬁcation	19 (9.5)	43 (4.7)	0.012	0.187	10.9 (5.8)	50.9 (5.5)	0.883	0.01
Lesion with aneurysm	0 (0.0)	5 (0.5)	0.642	0.105	0.0 (0.0)	4.1 (0.4)	0.307	0.095
ACC/AHA lesion classiﬁcation			0.041	0.232			0.454	0.14
Type A	17 (8.5)	130 (14.2)			19.2 (10.2)	123.3 (13.4)		
Type B1	72 (35.8)	366 (39.9)			71.9 (38.1)	357.7 (38.9)		
Type B2	70 (34.8)	254 (27.7)			62.7 (33.2)	256.3 (27.8)		
Type C	42 (20.9)	168 (18.3)			34.9 (18.5)	183.1 (19.9)		
Pre-procedure TIMI ﬂow			0.620	0.076			0.559	0.053
TIMI 2 flow	1 (0.5)	11 (1.2)			1.1 (0.6)	9.8 (1.1)		
TIMI 3 flow	200 (99.5)	907 (98.8)			187.5 (99.4)	910.7 (98.9)		
Reference vessel diameter, mm	2.92 ± 0.34	2.92 ± 0.34	0.929	0.007	2.90 ± 0.36	2.92 ± 0.34	0.537	0.056
Intravascular imaging using	18 (9.0)	65 (7.1)	0.441	0.069	13.8 (7.3)	69.4 (7.5)	0.916	0.008
Pre-PCI FFR	0.76 ± 0.06	0.81 ± 0.11	<0.001	0.527	0.78 ± 0.06	0.80 ± 0.11	<0.001	0.26

Values are mean ± SD or *n* (%).

ACC, American College of Cardiology; AHA, American Heart Association; FFR, fractional ﬂow reserve; IPTW, inverse-probability-treatment weighting; TIMI, Thrombolysis In Myocardial Infarction.

### Distribution of FFR and diameter stenosis

**Figure 2:**
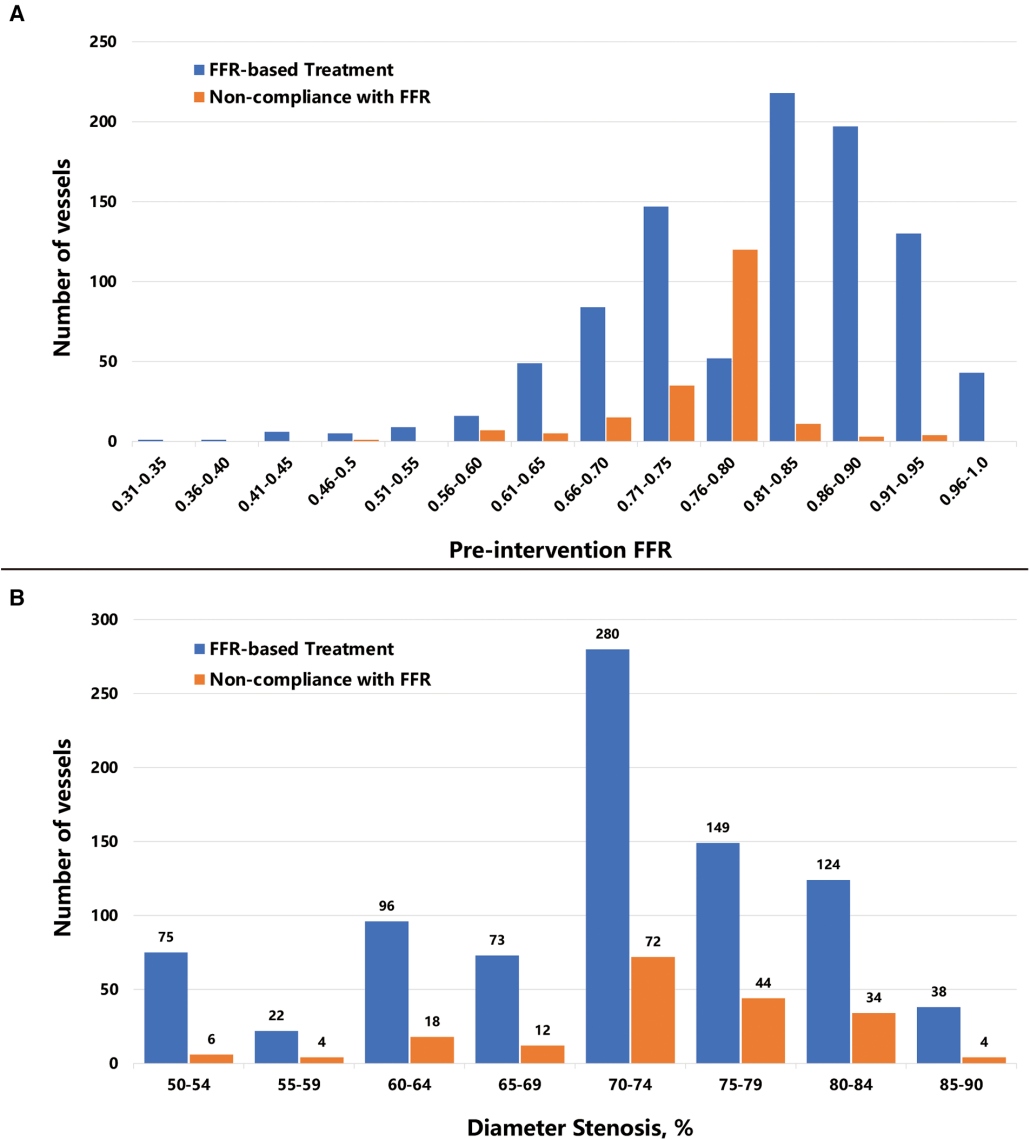
Distribution of pre-intervention FFR values and diameter stenosis. The bar plot shows (**A**) the number of vessels distributed per 0.05 FFR increment, and (**B**) the number of vessels distributed per 5% diameter stenosis increment. Abbreviations as in Figure 1.

Most of the investigated vessels exhibited an FFR of between 0.66 and 0.95, presenting a left-skewed distribution. Vessels for which treatment decisions were not compliant with the FFR results were primarily situated within the 0.56–0.80 range (Figure 2A). More than 80% of vessels had 60%–84% stenosis, while non-compliant vessels are mainly concentrated in the stenosis of 70%–84% (Figure 2B).

### Clinical follow-up outcomes

**Figure 3:**
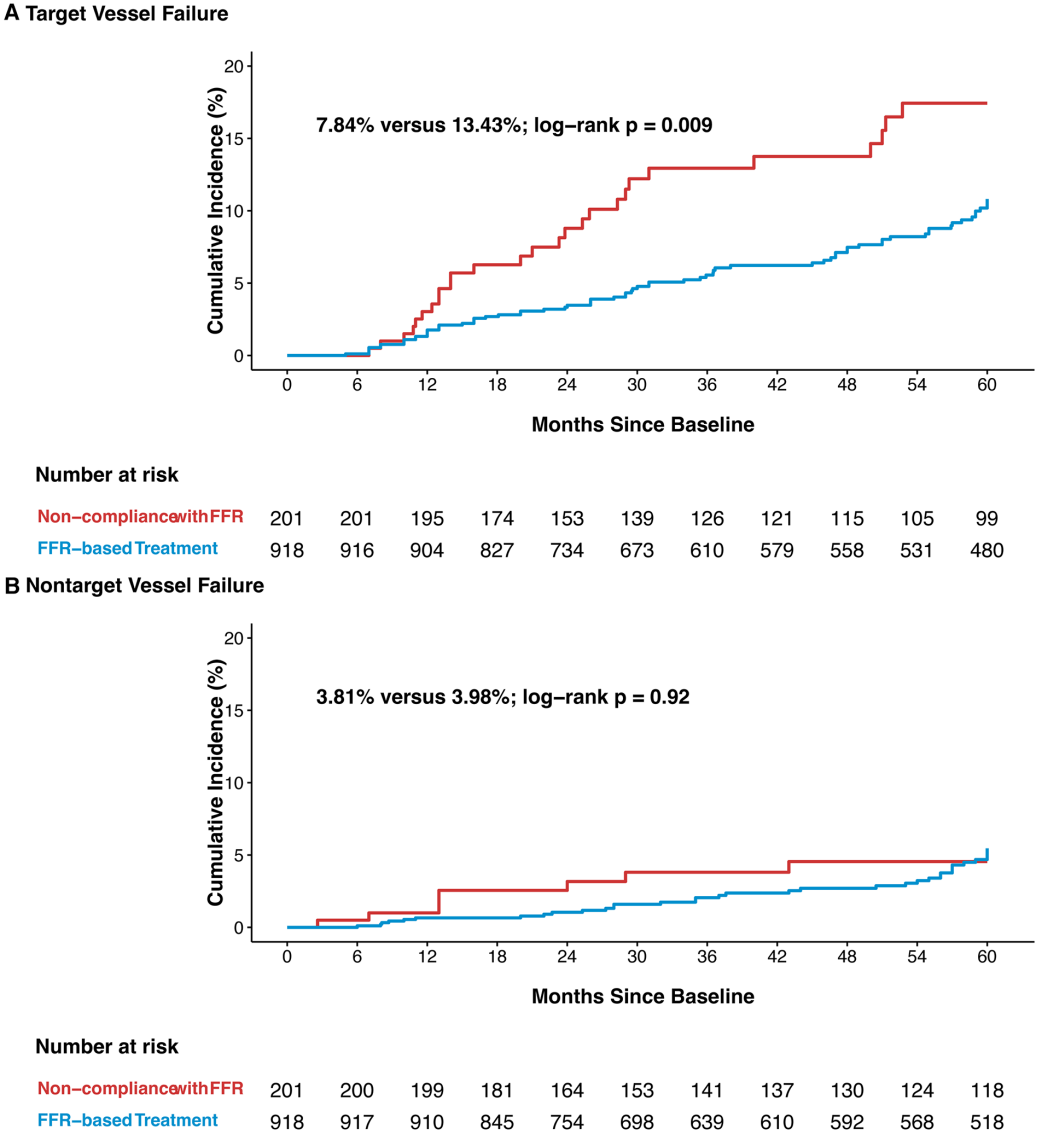
Kaplan-Meier curves of target vessel failure (**A**), and nontarget vessel failure (**B**) TVF, target vessel failure; NTVF, nontarget vessel failure; other abbreviations as in Figure 1.

The vessel-oriented events observed during the median follow-up of 58 months (interquartile range: 27–60 months) are detailed in Figure 3. Of the 139 observed vessel-related events, 71.2% occurred in the vessels assessed by FFR (i.e., TVF). TVF was observed in 72 vessels treated with FFR guidance, in contrast to 27 vessels among non-compliance group (7.84% vs. 13.43%; *p* = 0.009). The incidence of non-target vessel failure (NTVF) was comparable between FFR-based group and non-compliance group, standing at 35 vessels (3.81%) vs. 8 vessels (3.98%) (*p* = 0.920).

In IPTW-adjusted multivariate analysis, a lower risk for TVF was observed in the FFR-based treatment group than in the non-compliance with FFR group (adjusted HR: 0.56; 95% CI: 0.34–0.92). In accordance with the individual clinical outcome components listed in Table 3, the inter-group differences in TVF were mainly driven by a lower incidence of TVR (adjusted HR: 0.56; 95% CI: 0.33–0.93). There was no significant difference in the incidence of NTVF between vessels in the FFR-based group and the non-compliance group (adjusted HR: 1.02; 95% CI: 0.45–2.31). Furthermore, FFR-based treatment did not show an independent correlation with the reduced risk of VOCEs (adjusted HR: 0.69; 95% CI: 0.45–1.05) (Table 3).

**Table 3:** Clinical outcomes at 5-year follow-up.

		Compliance with FFR		Hazard ratio (95% CI)	
		No (*n* = 201)	Yes (*n* = 918)	Multivariate analysis^a^	Adjusted cox model with IPTW
TVF		27 (13.43)	72 (7.84)	0.58 (0.36–0.94)	0.56 (0.34–0.92)
	TVD	0 (0.00)	4 (0.44)	-	-
	TVMI	5 (2.49)	7 (0.76)	0.23 (0.06–0.95)	0.21 (0.05–0.86)
	TVR	26 (12.94)	67 (7.30)	0.57 (0.35–0.94)	0.56 (0.33–0.93)
NTVF		8 (3.98)	35 (3.81)	1.14 (0.52–2.50)	1.02 (0.45–2.31)
	NTVMI	2 (1.00)	3 (0.33)	0.34 (0.04–2.64)	0.29 (0.04–2.17)
	NTVR	6 (2.99)	35 (3.81)	1.55 (0.64–3.75)	1.53 (0.59–3.96)
VOCEs^b^		34 (16.92)	105 (11.44)	0.71 (0.47–1.07)	0.69 (0.45–1.05)

Values are *n* (%).

CI, conﬁdence interval; NTVF, nontarget vessel failure; NTVMI, nontarget vessel myocardial infarction; NTVR, nontarget vessel revascularization; TVMI, target vessel myocardial infarction; TVD, target vessel death; TVF, target vessel failure; TVR, target vessel revascularization; VOCEs, vessel-oriented composite endpoints; other abbreviations as in Table 2.

^a^ Included covariates were age, BMI, current smoking, diabetes mellitus, hypertension, dyslipidemia, previous myocardial infarction, previous percutaneous coronary intervention, previous cerebrovascular disease, peripheral artery disease, clinical indication, left ventricular ejection fraction, target vessel, multivessel disease, diameter stenosis, fractional flow reserve, ACC/AHA lesion classiﬁcation, bifurcation lesion and moderate-to-severe calciﬁcation.

^b^ Composite of cardiac death, any myocardial infarction and any unplanned revascularization.

Based on the landmark analysis, no differences in TVF were observed between the two groups (1.3% vs. 3.0%; *p* = 0.088) during the initial 12-month follow-up period. In the follow-up period extending from 12–60 months, the FFR-based treatment group exhibited a lower incidence of TVF compared to the non-compliance with FFR group (10.0% vs. 15.1%; *p* = 0.037) (Supplementary Figure S1). The rates of NTVF were comparable among the two groups during the first 12 months (0.7% vs. 1.0%; *p* = 0.6) and between 12 and 60 months (5.2% vs. 3.8%; *p* = 0.89) (Supplementary Figure S2).

### Subgroup analyses

**Figure 4:**
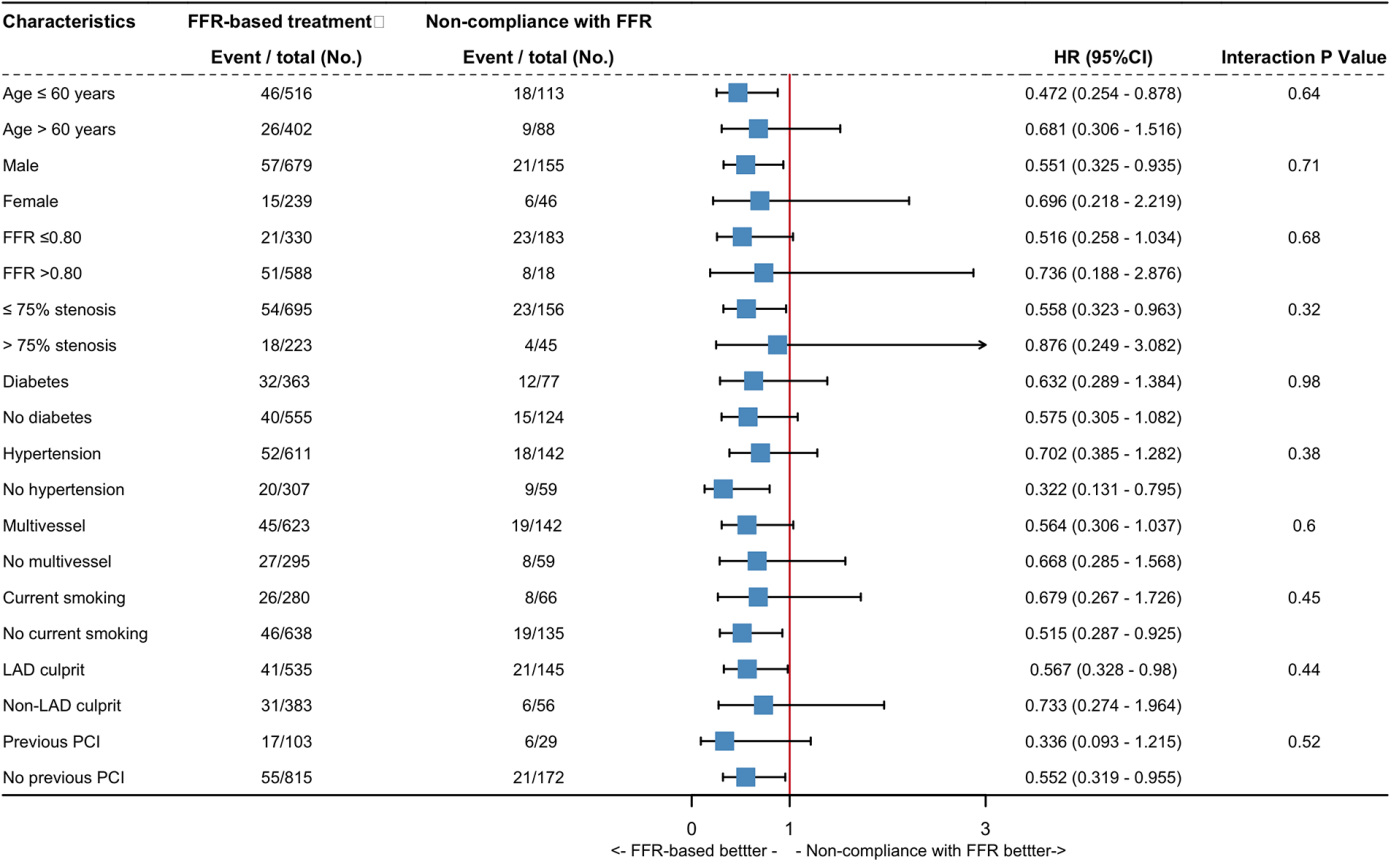
Exploratory subgroup analysis for target vessel failure. Hazard ratio estimates for the TVF are shown with their 95% conﬁdence interval, according to the various subgroups. CI, conﬁdence interval; HR, hazard ratio; LAD, left anterior descending artery, other abbreviations as in Figure 1.

Subgroup analyses on TVF and NTVF were performed across ten subgroups, categorized based on prognostically-relevant baseline statuses (Figure 4, Supplementary Figure S3). Within vessels exhibiting an FFR of 0.80 or less, a trend towards decreased TVF rates was detected in the FFR-based group, compared with the non-compliance with FFR group (HR: 0.52; 95% CI: 0.26–1.03; *p* for interaction = 0.68). However, this trend was not mirrored in the rates of NTVF (HR: 1.54; 95% CI: 0.62–3.84; *p* for interaction = 0.16). Among vessels demonstrating ≤75% diameter stenosis, FFR-based treatment was associated a significantly lower hazard of TVF in comparison with the non-adherence approach (HR: 0.56; 95% CI:0.32–0.96; *P* interaction = 0.32). In vessels from patients with multivessel disease, subgroup comparisons revealed a trend toward a lower rate of TVF in the FFR-based group compared to the non-compliance with FFR group (HR: 0.56; 95% CI: 0.31–1.04; *p* for interaction = 0.6). Nevertheless, this trend was not observed with respect to NTVF (HR: 1.25; 95% CI: 0.51–3.07; *p* for interaction = 0.53). No significant interaction was observed between baseline status and procedural variables. It is worth to noting that the subgroup analyses were underpowered; thus, the aforementioned conclusions should be interpreted as hypothesis-generating.

### Sensitivity analyses

Considering the potentially factors that might impact the precision of FFR measurements, a sensitivity analysis was conducted on the remaining 919 vessels after excluding vessels with ostial or tortuous lesions, TIMI flow grade of <3, and vessels from patients presented with acute MI. As demonstrated in Supplementary Table S1, FFR-based treatment was associated with a significantly lower hazard of TVF at the 5-year follow-up compared to the non-compliance FFR approach (8.61% vs. 14.02%; HR: 0.56; 95% CI: 0.32–0.96). In terms of NTVF, the results were generally aligned with the main findings. In this cohort, there were 902 vessels with stenosis between 60% and 84%, representing over 80% of the total. Within this range, FFR-based treatment was correlated with a significantly lower hazard of TVF at 5 years (7.76% vs. 13.89%; HR: 0.53; 95% CI: 0.31–0.90) when compared with non-compliance FFR group. Consistent with the main results, there was no statistically significant association between FFR-based treatment and NTVF (4.02% vs. 4.44%; HR: 0.99; 95% CI: 0.42–2.31) (Supplementary Table S2).

## Discussion

The findings of this study reveal that, across a broad range of patient demographics, a considerable proportion of angiographically significant vessels measured by FFR, did not receive FFR-based treatment in clinical practice. This non-adherence to FFR is linked with a higher rate of 5-year TVF, including cardiac death, TVMI, and unplanned TVR. On the contrary, no correlation was observed between non-compliance with FFR and NTVF. Additionally, upon further investigation into VOCEs, which encompassing the aforementioned types of vessel-related events, no definitive correlation was found between non-compliance with FFR and VOCEs. These findings validate the effectiveness of FFR in guiding revascularization within routine clinical practice, while raising queries concerning the appropriateness of conventional endpoint categorization in research that explores the clinical benefits of the FFR-based approach.

In the present study, a total of 201 vessels did not receive the FFR-based treatment strategy, representing 35% (*n* = 183) of vessels with ischemic FFR and 3% (*n* = 18) of vessels with non-ischemic FFR, respectively. The proportions of underutilization and overutilization of FFR-based revascularization in the present study corresponds with previous registries (12, 13).

A population-based study involving patients with CAD who underwent single-vessel FFR measurement examined the association between adherence to FFR treatment and major adverse cardiovascular events (MACEs) (6), and showed that PCI was significantly associated with a lower rate of MACEs in ischemic lesions and a higher rate of MACEs in non-ischemic lesions. Our findings on the prognostic benefits of FFR based methods extend the results of the aforementioned studies. Nonetheless, it is essential to acknowledge several differences between the two studies. First, in the population-based study, all of the included participants had only one vessel subjected to FFR measurement. Conversely, the present study did not impose any restrictions on the number of vessels subject to FFR measurement in the enrolled patients. Additionally, in the subgroup analysis concerning multivessel disease samples, a benefit on TVF in favor of the FFR-based approach was demonstrated. Secondly, the population-based study performed a patient-level analysis, with the primary outcomes being MACEs, including all-cause mortality, MI, unstable angina, and urgent revascularization. In contrast, we analyzed the endpoints on a per-vessel basis, defined as TVF, which included cardiac death, TVMI, and urgent TVR.

The primary objectives of coronary artery FFR assessment are to ascertain whether existing coronary artery lesions result in inadequate myocardial perfusion and to assess the need for target vessel revascularization to improve impaired myocardial perfusion. Employing FFR to guide decisions regarding the applying of PCI has been shown to reduce the potential risk of subsequent target vessel adverse events when compared with the use of angiographic guidance (4, 5) It is worth noting that, however, during the follow-up period of previous clinical trials on FFR, a noteworthy proportion of adverse events occurred in vessels that were not investigated by FFR (2, 14). Therefore, if the relative contribution of each endpoint categorization to the overall event rate is not clearly distinguished in such trials, it may affect the reliability of the conclusions. In the present study, a significant difference favoring the FFR-based approach was observed with respect to TVF. Nonetheless, when including overall vessel-oriented composite events in the analysis without distinguishing the endpoints between target and non-target vessels, this benefit was no longer apparent. Furthermore, as the follow-up period extends, the number of endpoints related to the specific cause studied could be diluted by the natural occurrence of endpoints originating from other causes. Confirmation of this perspective may be obtained from Fractional Flow Reserve Versus Angiography for Guiding Percutaneous Coronary Intervention (FAME) study. This multicenter randomized trial indicated that the superiority of the FFR-guided strategy is primarily observed in the early stage (4, 15). However, by the time of 5-year follow-up, the difference in MACEs rate favoring FFR guidance was attenuated (16). The results yielded from the landmark analysis of this study reveal no convergence of the TVF incidence between groups in the long term, which aligns with findings from other studies that regard target vessel events as research endpoints and conduct independent analyses (2, 17, 18). Nonetheless, the landmark analysis with respect to VOCEs in the present study demonstrated the occurrence of catch-up over time in the FFR-based group. The findings of this study indicate that when assessing the benefits of the FFR-based approach, it is essential to clearly categorize the endpoints (i.e., target vessel-related, non-target vessel-related, or overall). However, our analysis should be taken as hypothesis-generating, and further well-designed studies should be conducted to provide further insight into this issue.

### Study limitations

The findings from this study should be interpreted in light of the following limitations. First, as with other observational studies, the present analyses were also inevitably subjected to residual confounding and selection bias, although intergroups baseline differences were minimized by performing IPTW-adjustment. Second, a limited number of cardiac death events were observed in this cohort, which might be partly attributed to the remarkable proportion of participants that were censored during the 5-year follow-up period. However, we did not conduct the sensitivity analysis performed in the FAME study (16), which assumed that all lost-to-follow-up patients experienced death after their last visit. This is because attributing such a large number of death events arbitrarily to specific vessels might compromise the validity of the analytical conclusions. Finally, despite the differences in the subsequent risk of adverse events experienced by vessels with ischemic and non-ischemic FFR, we present the clinical outcomes of ischemic and non-ischemic vessels separately, only in the subgroup analyses. The limited number of events among the ischemic group (FFR ≤ 0.8) and non-ischemic group (FFR > 0.8) do not allow a meaningful conclusion to be drawn. However, a trend favoring the FFR-based approach was observed in the ischemic group.

## Conclusions

Deviations from established threshold in applying FFR within routine clinical practice are not infrequent, with numerous potential lesion characteristics correlated with this occurrence. In patients with coronary artery disease subjected to FFR, the FFR-based treatment yields a sustained clinical benefit, as compare with non-compliance with FFR in terms of the risks of target vessel-related events. The dilution of non-target vessel-related events renders the difference favoring the FFR-based approach nonsignificant.

## Data Availability

The raw data supporting the conclusions of this article will be made available by the authors, without undue reservation.
