# Supplementary material for: Prognostic significance of compliance with fractional flow reserve guidance on diverse vessel-related clinical outcomes
Source: Front Cardiovasc Med. 2024 May 17;11:1370345. doi: 10.3389/fcvm.2024.1370345 (PMC11140391; doi:10.3389/fcvm.2024.1370345)
Supplement: Supplementary file 1 [file Datasheet1.docx]

**Table S1. Clinical Outcomes of Vessel without Myocardial Infarction, Ostial Lesion, Tortuous Lesion, or TIMI<3.**

|  | **Compliance with FFR** | | **Hazard ratio (95% CI)** | |
| --- | --- | --- | --- | --- |
|  | **No (n = 164)** | **Yes (n = 755)** | **Multivariate analysis** † | **Adjusted Cox Model with IPTW** |
| **TVF** | 23 (14.02) | 65 (8.61) | 0.57 (0.33 - 0.97) | 0.56 (0.32 - 0.96) |
| **NTVF** | 7 (4.27) | 28 (3.71) | 1.12 (0.47 - 2.67) | 1.03 (0.41 - 2.61) |
| **VOCEs** ‡ | 30 (18.29) | 92 (12.19) | 0.66 (0.42 - 1.04) | 0.65 (0.41 - 1.03) |

Values are n (%). † Included covariates were age, BMI, current smoking, diabetes mellitus, hypertension, dyslipidemia, previous myocardial infarction, previous percutaneous coronary intervention, previous cerebrovascular disease, peripheral artery disease, clinical indication, left ventricular ejection fraction, target vessel, multivessel disease, diameter stenosis, fractional flow reserve, ACC/AHA lesion classiﬁcation, bifurcation lesion and moderate-to-severe calciﬁcation. ‡ Composite of cardiac death, any myocardial infarction and any unplanned revascularization. CI = conﬁdence interval; IPTW = inverse-probability-treatment weighting; FFR = fractional ﬂow reserve; NTVF = nontarget vessel failure; TVF = target vessel failure; TVR = target vessel revascularization; VOCEs = vessel-oriented composite endpoints; other abbreviations as in Table 1.
