# Supplementary figures and images for: Prognostic significance of compliance with fractional flow reserve guidance on diverse vessel-related clinical outcomes
Source: Front Cardiovasc Med. 2024 May 17;11:1370345. doi: 10.3389/fcvm.2024.1370345 (PMC11140391; doi:10.3389/fcvm.2024.1370345)

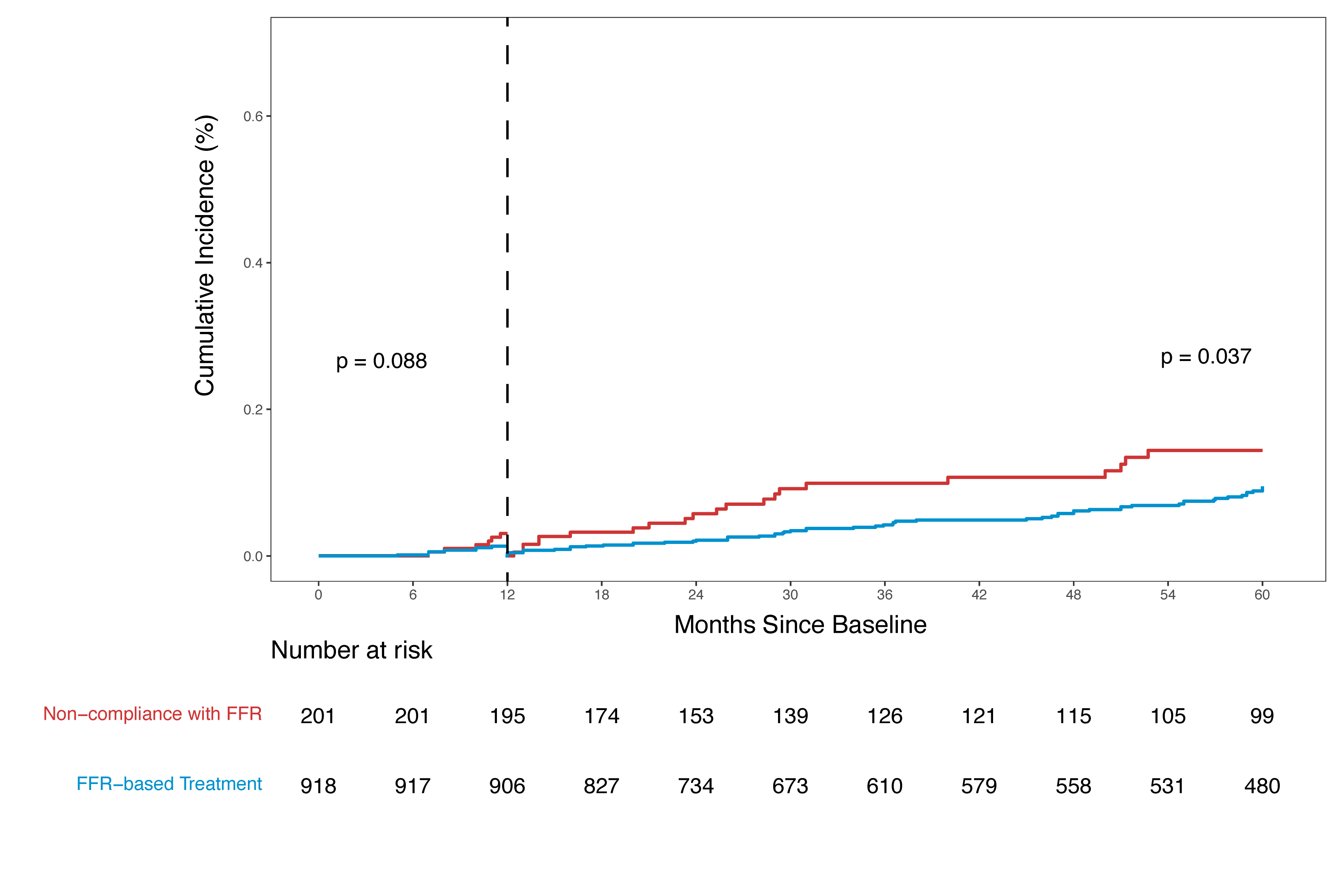

Supplement: Supplementary file 3 [file Image1.tif]

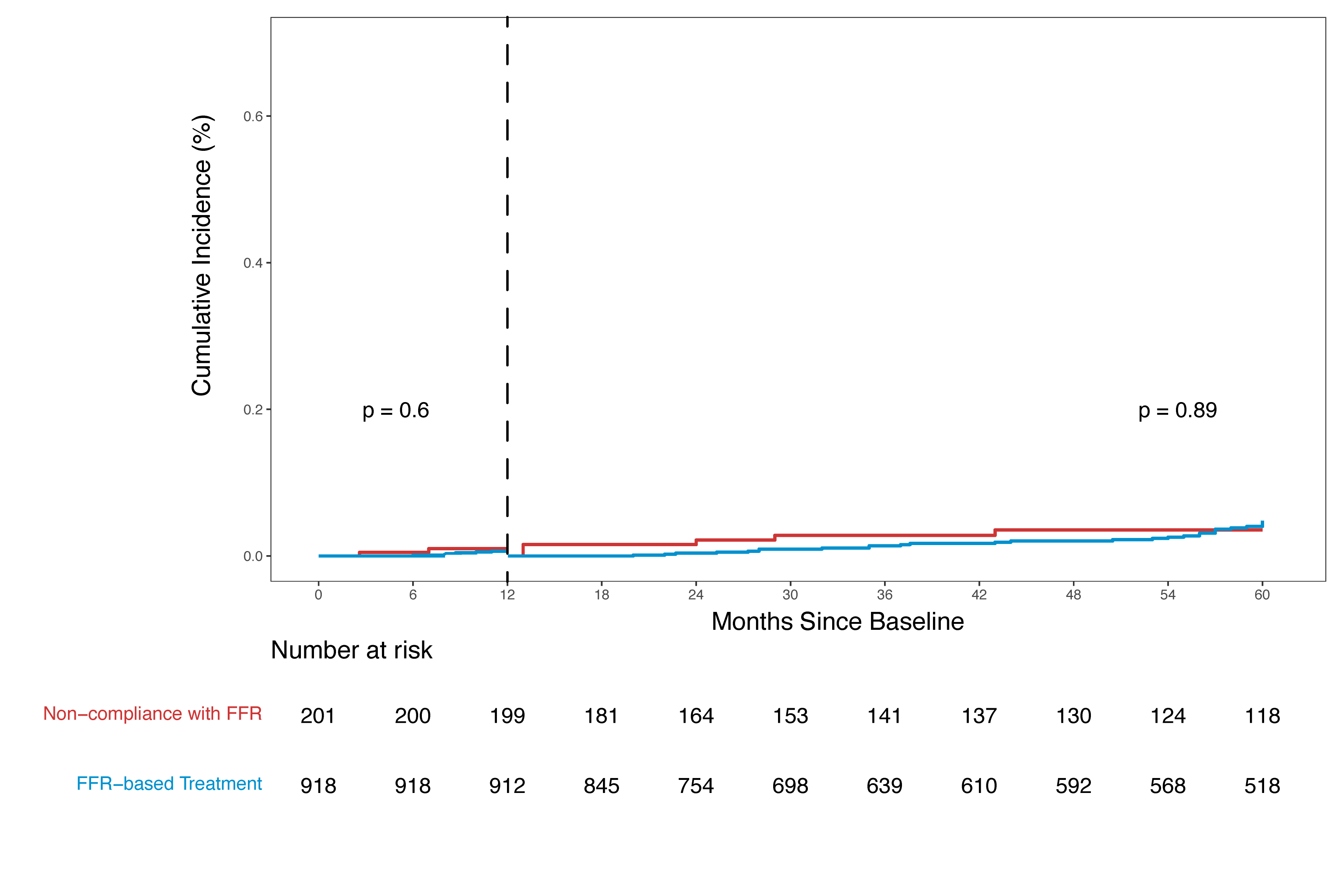

Supplement: Supplementary file 4 [file Image2.tif]

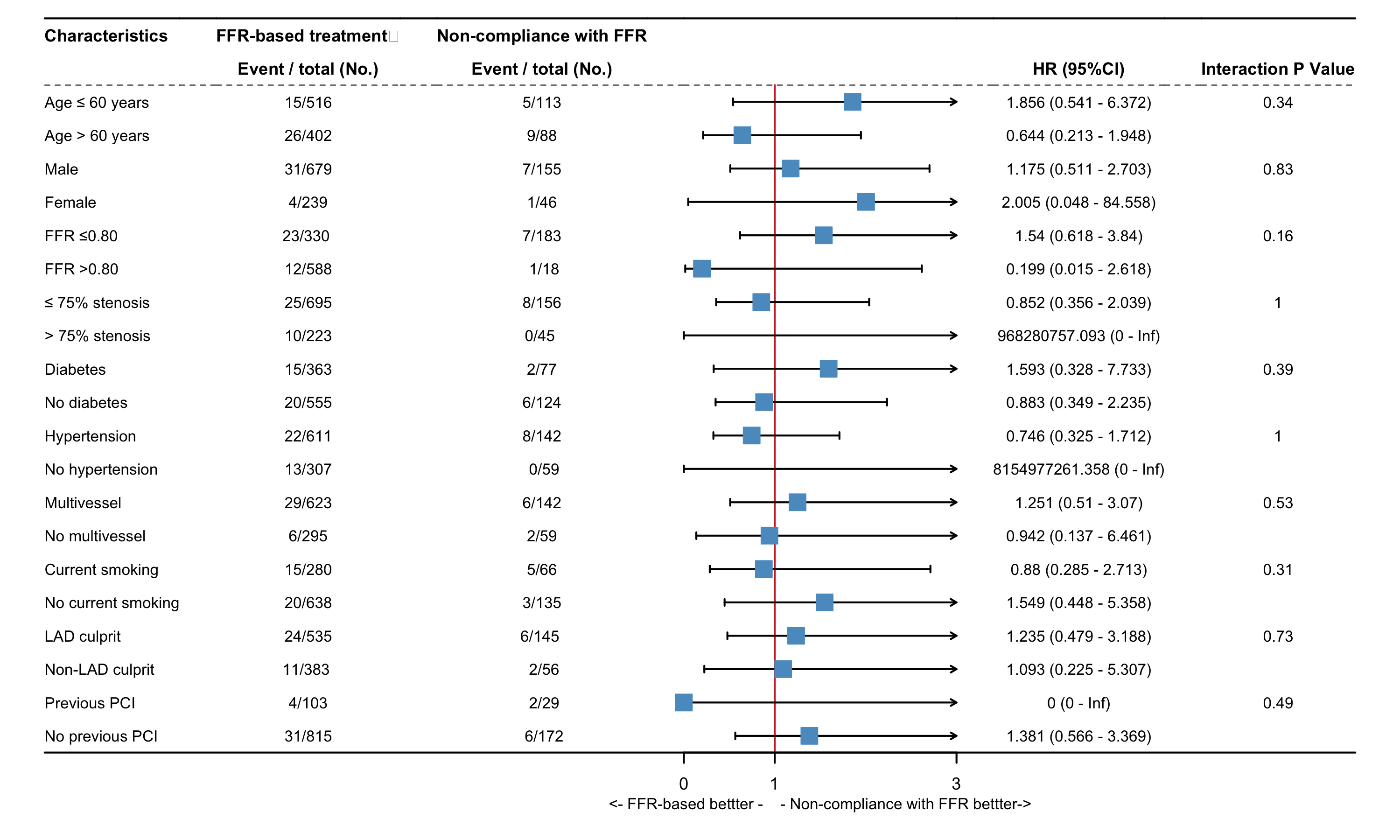

Supplement: Supplementary file 5 [file Image3.tiff]
